# Interaction of standardized mistletoe (*Viscum album*) extracts with chemotherapeutic drugs regarding cytostatic and cytotoxic effects *in vitro*

**DOI:** 10.1186/1472-6882-14-6

**Published:** 2014-01-08

**Authors:** Ulrike Weissenstein, Matthias Kunz, Konrad Urech, Stephan Baumgartner

**Affiliations:** 1 Society for Cancer Research, Hiscia Institute, Arlesheim, Switzerland; 2 Institute of Integrative Medicine, Witten/Herdecke University, Herdecke, Germany

**Keywords:** Mistletoe (*Viscum album* L.), Iscador, Chemotherapy, Drug interactions, Cytostasis, Cytotoxicity

## Abstract

**Background:**

Given the importance of complementary and alternative medicine (CAM) to cancer patients, there is an increasing need to learn more about possible interactions between CAM and anticancer drugs. Mistletoe (*Viscum album* L.) belongs to the medicinal herbs that are used as supportive care during chemotherapy. In the *in vitro* study presented here the effect of standardized mistletoe preparations on the cytostatic and cytotoxic activity of several common conventional chemotherapeutic drugs was investigated using different cancer cell lines.

**Methods:**

Human breast carcinoma cell lines HCC1937 and HCC1143 were treated with doxorubicin hydrochloride, pancreas adenocarcinoma cell line PA-TU-8902 with gemcitabine hydrochloride, prostate carcinoma cell line DU145 with docetaxel and mitoxantrone hydrochloride and lung carcinoma cell line NCI-H460 was treated with docetaxel and cisplatin. Each dose of the respective chemotherapeutic drug was combined with *Viscum album* extract (VAE) in clinically relevant concentrations and proliferation and apoptosis were measured.

**Results:**

VAE did not inhibit chemotherapy induced cytostasis and cytotoxicity in any of our experimental settings. At higher concentrations VAE showed an additive inhibitory effect.

**Conclusions:**

Our in vitro results suggest that no risk of safety by herb drug interactions has to be expected from the exposition of cancer cells to chemotherapeutic drugs and VAE simultaneously.

## Background

A primary cytotoxic mechanism of many conventional anticancer agents is based on the damage of DNA and the subsequent induction of apoptosis. Beside cytotoxic reactions cancer cells can also respond by cell cycle block or delay (cytostasis) [1]. Because chemotherapeutic agents preferably act on rapidly dividing normal cells, therapeutic treatments result in common side-effects like myelosuppression, hair loss, fatigue, infection etc. In an attempt to reduce the clinical toxicity of chemotherapeutic drugs, to consolidate the immune system and to improve the symptoms of their disease many cancer patients use mistletoe extracts as a complementary therapy in combination with standard regimens [2,3].

Mistletoe (*Viscum album*) preparations contain active components like mistletoe lectins and viscotoxins and are reported to show anti-tumoral properties by causing cell cycle delay or arrest and induction of apoptosis [4-7], affecting tumor angiogenesis [8,9] and exerting immune-potentiating activities that may enhance the host defense system against tumors [10-13]. Molecular compounds of mistletoe are reported to show in vitro inhibitory potential on P-glycoprotein (P-gp) also known as multidrug resistance protein 1 (MDR1) [14]. The analysis of clinical studies suggests that adjuvant treatment of cancer patients with mistletoe extracts is associated with a better survival, a reduction of side effects of conventional therapy and with an increase of quality of life [15-18]. In early stage breast cancer patients the frequency of relapse or metastasis within 5 years was not influenced by additional mistletoe therapy [19].

Oncologists, confronted with the decision of their patients to use complementary therapies, sometimes are concerned about possible interactions of herbal medicines with oncological drugs, which could influence the efficacy of the standard treatment.

The aim of our study therefore was to investigate possible effects of clinically relevant doses of standardized VAEs on the cytostatic and cytotoxic efficacy of several standard chemotherapeutic agents on different cancer cell lines *in vitro*.

## Methods

### Mistletoe extracts and chemotherapeutic drugs

The aqueous, fermented mistletoe preparations Iscador M spec. 5 mg (VAE-M, host tree *Malus domestica*, Lot 1109/2103/2, total mistletoe lectin concentration 287 ng/ml) and Iscador Qu spec. 5 mg (VAE-Qu, host tree *Quercus robur* and *Q. petraea*, Lot 1204/2221/4, total mistletoe lectin concentration 399 ng/ml) were obtained from the Society for Cancer Research (Arlesheim, Switzerland).

Doxorubicin hydrochloride, gemcitabine hydrochloride, docetaxel, and mitoxantrone hydrochloride were obtained from Sigma-Aldrich Logistik GmbH (Buchs, CH) and cisplatin from LuBio Science GmbH (Lucerne, CH).

### Cell culture

Human breast carcinoma cell lines HCC1937 and HCC1143, pancreas adenocarcinoma cell line PA-TU-8902, prostate carcinoma cell line DU145 and lung carcinoma cell line NCI-H460 were obtained from DSMZ (German Collection of Microorganisms and Cell Cultures, Braunschweig, Germany).

HCC1937, HCC1143, DU145 and NCI-H460 cells were cultured in RPMI-1640 supplemented with 10% fetal calf serum, 2 mM L-glutamine, and 1% Penicillin – Streptomycin (Sigma-Aldrich). PA-TU-8902 cells were cultured in Dulbecco’s MEM High Glucose (Sigma-Aldrich) supplemented with 2 mM L-Glutamine, 1 mM Sodium Pyruvate, 10% fetal calf serum and 1% Penicillin – Streptomycin in a humidified atmosphere with 5% CO_2_ at 37°C. Cell lines were maintained in exponential growth and cells from subconfluent monolayers were harvested by trypsin-EDTA (Sigma-Aldrich) to carry out the experiments. For measurement of the parameters, the cell cultures were used within 4–6 weeks after thawing.

### Proliferation assay

Proliferation was indirectly assessed using the cell proliferation reagent WST-1 (Roche, Mannheim, Germany). Cells (1.5 × 10^4^ in 100 μl) were plated in triplicates in 96-well plates. After 4–6 hours to allow attachment, the drugs were added in various concentrations (see below). Proliferation rate was measured 4 h after incubation with the reagent in triplicate. The upper limit of absorbance was 2.0 - 2.1. Values are given in percent inhibition of proliferation relative to untreated control.

### Cell death analysis

Apoptosis/necrosis was measured using the Annexin V-FITC Apoptosis Detection Kit I (BD Biosciences Pharmingen™, San Diego, CA, USA). Briefly: 2x10^5^ cells were incubated with Annexin V-FITC and 7-AAD at room temperature in the dark. Thereafter, the samples were analysed in a flow cytometer (FACS Calibur, BD Biosciences, San Jose, CA). Early apoptotic cells: Annexin V-FITC positive and 7-AAD negative. Late apoptotic/necrotic cells: Annexin V-FITC positive and 7-AAD positive. Values are given in percent of total cell number. Cytotoxicity (%) was calculated as follows: early apoptotic cells (%) + late apoptotic/necrotic cells (%).

### Drug concentrations in the assays

Preceding the actual experiments the dose–response concentration range and the optimal incubation time was determined for each chemotherapeutic agent and each cell line individually using the WST-1 proliferation assay (data not shown). Cells were incubated for 48 h or 72 h respectively, depending on the maximal measurable anti-proliferative effect of cytostatic agents. Because of its own fluorescence, doxorubicin at higher doses interfered with the nucleic acid dye 7-AAD. Therefore the maximal doxorubicin concentration usable for the detection of apoptosis in the breast carcinoma cell lines HCC1143 and HCC1937 was 5 μg/ml.

In the main experiments, the drugs were added in culture medium at the concentrations indicated in Table 1. Each dose of the respective chemotherapeutic drug was combined with VAE-M (HCC1937 and HCC1143) or VAE-Qu (PA-TU-8902, DU-145 and NCI-H460) at the concentrations of 0; 0.1; 1.0; 10; 100 μg/ml for the measurement of proliferation and of 0; 0.1; 1.0; 10 μg/ml for the measurement of apoptosis/necrosis. Typical clinical Iscador concentrations for subcutaneous application are 0.1 and 1 μg/ml, roughly corresponding to an injection of 5 mg Iscador when referring to the amount of circulating blood or body weight, respectively. Parameters were measured after the appropriate incubation time.

**Table 1:** Cell lines, chemotherapeutic drug concentrations and incubation times used for the proliferation and apoptosis/necrosis assays

**Cell line**	**Chemotherapeutic drug**	**Concentrations of chemotherapeutic drug (μg/ml) for**		**Incubation time (h)**
		**Proliferation assay**	**Apoptosis assay**	
HCC1937	Doxorubicin hydroxide	0.0; 0.1; 0.5; 1.0; 5.0	0.0; 0.25; 1.0; 5.0	48
HCC1143	Doxorubicin hydroxide	0.0; 0.1; 0.5; 1.0; 5.0	0.0; 0.5; 5.0	48
PA-TU-8902	Gemcitabine hydroxide	0.0; 0.01; 0.1; 1.0; 10.0	0.0; 25.0; 50.0; 100.0; 200.0	72
DU-145	Docetaxel	0.0; 0.0008; 0.008; 0.08; 0.8	0.0; 0.0008; 0.008; 0.08; 0.8	72
DU-145	Mitoxantrone hydroxide	0.0; 0.002; 0,02; 0,2; 2.0	0.0; 0.002; 0,02; 0,2; 2.0	72
NCI-H460	Cisplatin	0.0; 0.3; 1.0; 3.0; 9.0	0.0; 0.3; 1.0; 3.0; 9.0	48
NCI-H460	Docetaxel	0.0; 0.001; 0.01; 0.1; 1.0	0.0; 0.001; 0.01; 0.1; 1.0	48

As we intended to detect a minimal dose able to induce apoptosis in PA-TU-8902 cells we used considerably higher gemcitabine concentrations in apoptosis than in proliferation assay.

### Data analysis

Three independent experiments were carried out for each combination of chemotherapeutic drug and mistletoe extract. Data were analyzed with 2-way analysis of variance (ANOVA, Type 6 decomposition) using Statistica 6.0 (Statsoft Inc., Tulsa, USA). For pairwise comparisons, the protected Fisher LSD-test was used. This procedure gives a good safeguard against false-positive as well as false-negative errors [20]. Limit of significance was defined as *p* < 0.05.

## Results

### Effects of VAE on proliferation and apoptosis of cancer cell lines

**Figure 1:**
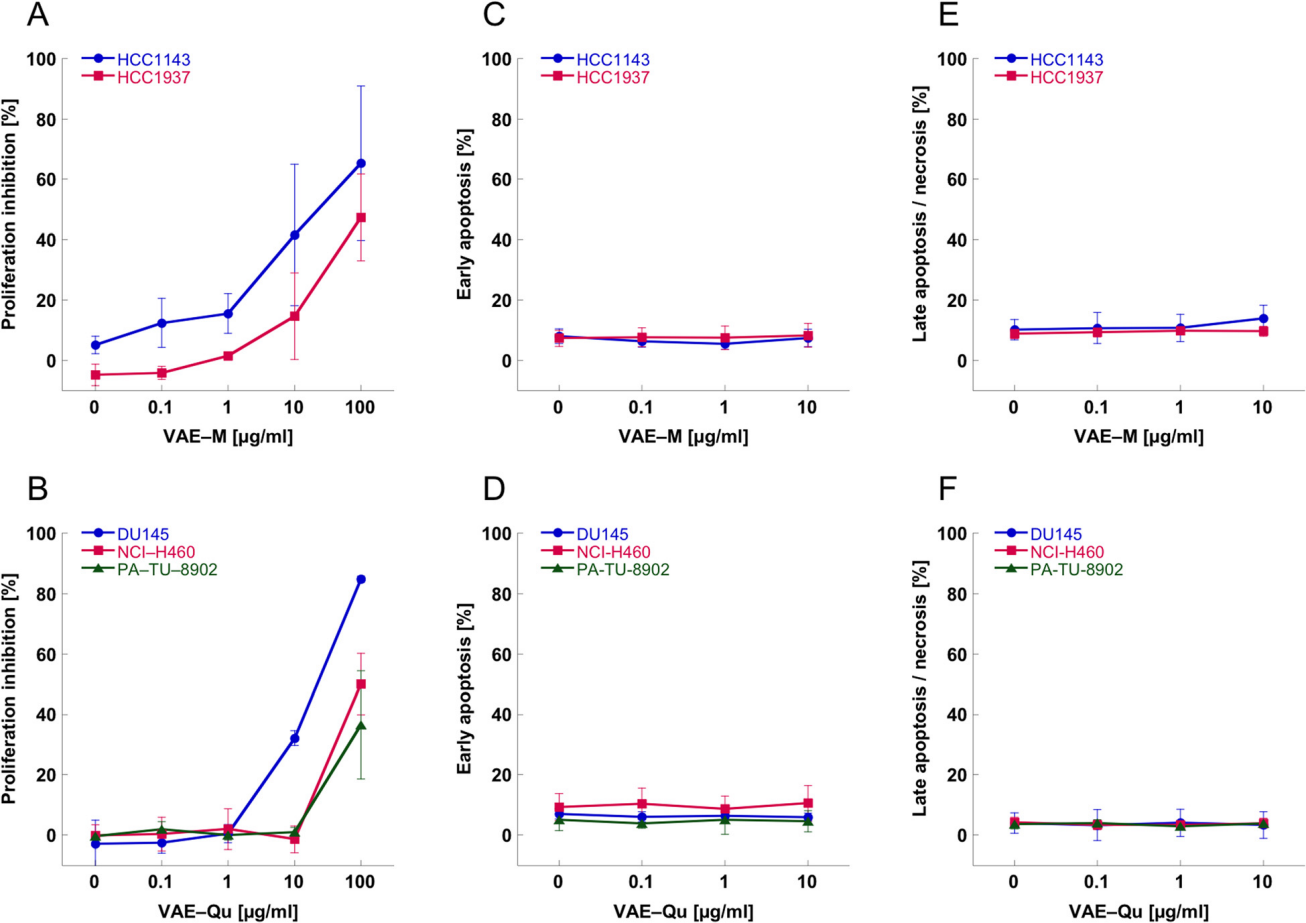
**Dose dependent anti-proliferative effect of A) VAE-M and B) VAE-Q and effect of VAE-M on C) early and E) late apoptosis/necrosis and of VAE-Qu on D) early and F) late apoptosis/necrosis of five cancer cell lines.** Results are presented as mean ± SD from three independent experiments. Cell growth kinetic was assessed with the WST-1 assay. Apoptosis was measured by flow cytometric analysis of Annexin V-FITC and 7-AAD double-labeled cells.

The growth kinetic analysis of five cancer cell lines revealed a dose dependent anti-proliferative effect of VAE at concentrations ≥10 μg/ml except for the pancreas carcinoma cell line PA-TU-8902 and the lung carcinoma cell line NCI-H460, where a proliferation inhibition could only be detected with 100 μg/ml (p < 0.05). The doses of 0.1 and 1 μg/ml VAE did not significantly influence the proliferation of tumor cells (Figure 1A,B). In all five cell lines (after 48 or 72 hours incubation, see Table 1) VAE concentrations between 0.1 and 10 μg/ml did not result in an elevated proportion of apoptotic and necrotic cells (p > 0.5, Figure 1C-F).

### Effects of a combined application of VAE and chemotherapeutic drugs on proliferation and apoptosis/necrosis in cancer cells

**Figure 2:**
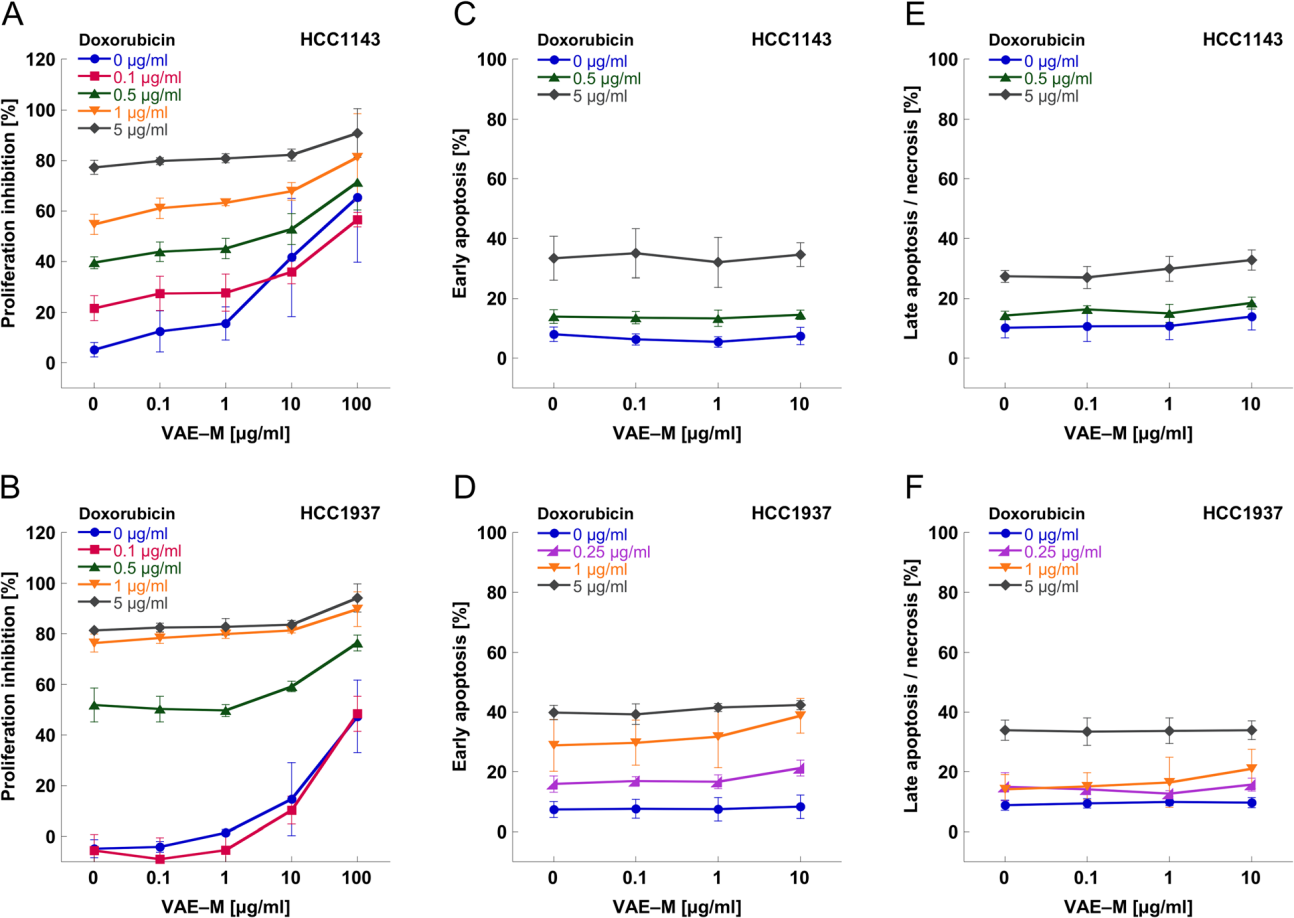
Mean values (±SD) of (A,B) proliferation, (C,D) early apoptosis and (E,F) late apoptosis/necrosis of breast carcinoma cell lines HCC1143 (A,C,E) and HCC1937 (B,D,F) treated with different concentrations of doxorubicin combined with different concentrations of VAE-M.

Figure 2 presents the mean values of proliferation, early apoptosis and late apoptosis/necrosis of the breast carcinoma cell lines HCC1143 and HCC1937 treated with different concentrations of doxorubicin in combination with different concentrations of VAE-M.

For HCC1143, the maximal cytostatic effect attained by the treatment with doxorubicin or VAE-M alone was about 75% or 65%, respectively. VAE-M generally enforced the antiproliferative effect of doxorubicin (Figure 2A). This enforcement was significant for 100 μg/ml VAE-M, compared to 0 μg/ml VAE-M, for the doxorubicin concentrations of 0.1–1 μg/ml (p < 0.01).

For HCC1937, the maximal cytostatic effect attained by the treatment with doxorubicin or VAE-M alone was about 80% or 45%, respectively. VAE-M ≥10 μg/ml enforced the antiproliferative effect of doxorubicin (Figure 2B). This enforcement was significant for 100 μg/ml VAE-M, compared to 0 μg/ml VAE-M, for all doxorubicin concentrations applied (p < 0.05).

A trend for an enhancement of the anti-proliferative effect of doxorubicin by VAE-M at the clinical relevant concentrations 0.1 and 1 μg/ml could be observed in the HCC1143 cell line, but not in HCC1937. This enforcement was not statistically significant.

According to the apoptosis measurements, doxorubicin exerted a dose dependent cytotoxic effect on HCC1143 and HCC1937 cells (p < 0.001). Maximal cytotoxicity measured was 60% and 75%, respectively. VAE-M at concentrations between 0.1 and 10 μg/ml neither induced cytotoxic effects nor influenced the cytotoxic effect of doxorubicin in both cell lines (p > 0.05) (Figure 2C,E,D,F).

**Figure 3:**
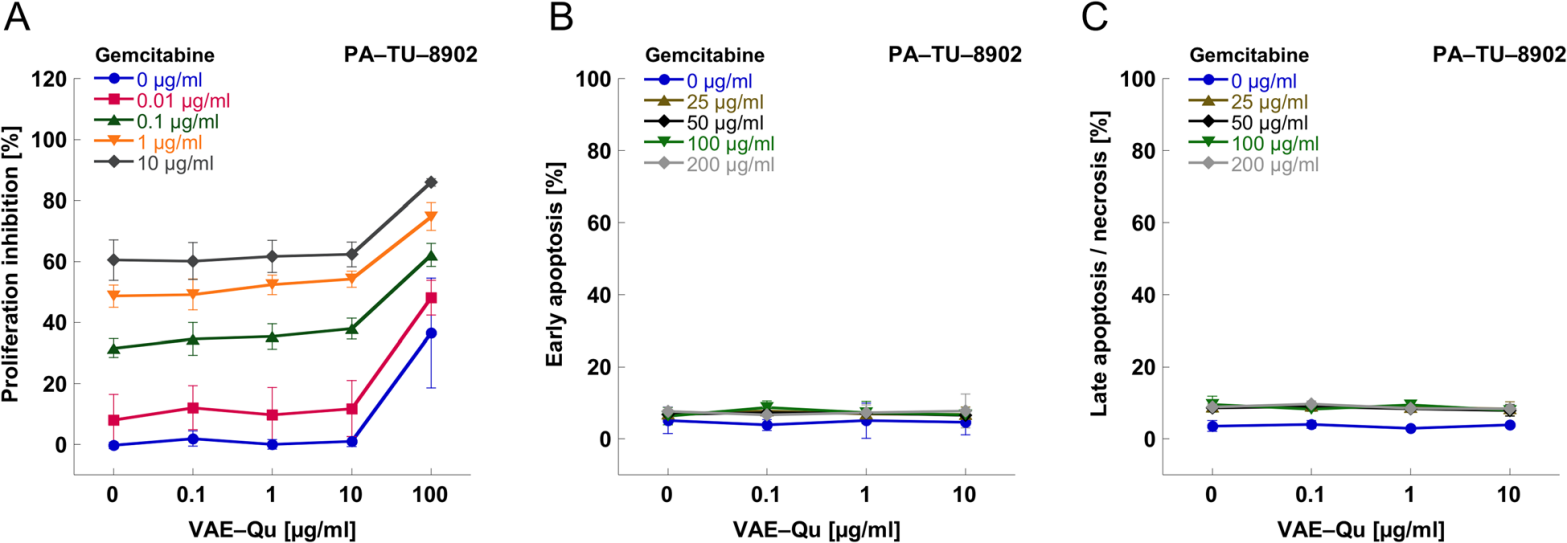
Mean values (±SD) of (A) proliferation, (B) early apoptosis and (C) late apoptosis/necrosis of pancreatic carcinoma cell line PA-TU-8902 treated with different concentrations of gemcitabine combined with different concentrations of VAE-Qu.

In the pancreatic carcinoma cell line PA-TU-8902 the maximal inhibition of proliferation attained by the treatment with 10 μg/ml gemcitabine or 100 μg/ml VAE-Qu alone was about 60% or 35%, respectively. Proliferation inhibition through gemcitabine could not be augmented further by dose enhancement of gemcitabine (data not shown). Only VAE-Qu at a concentration of 100 μg/ml resulted in an additional increase of the antiproliferative effect (p < 0.001) compared to VAE-Qu = 0 μg/ml for all gemcitabine concentrations. The pancreatic carcinoma cell line PA-TU-8902 was strongly apoptosis resistant. In this cell line the maximal cytotoxicity after 72 hours incubation was about 15% compared to 9% in the untreated control for all gemcitabine doses between 25 and 200 μg/ml and no concentration dependency was observed. VAE-Qu at concentrations between 0.1 and 10 μg/ml neither induced apoptosis nor influenced the cytotoxic effect of gemcitabine (p > 0.05, Figure 3).

**Figure 4:**
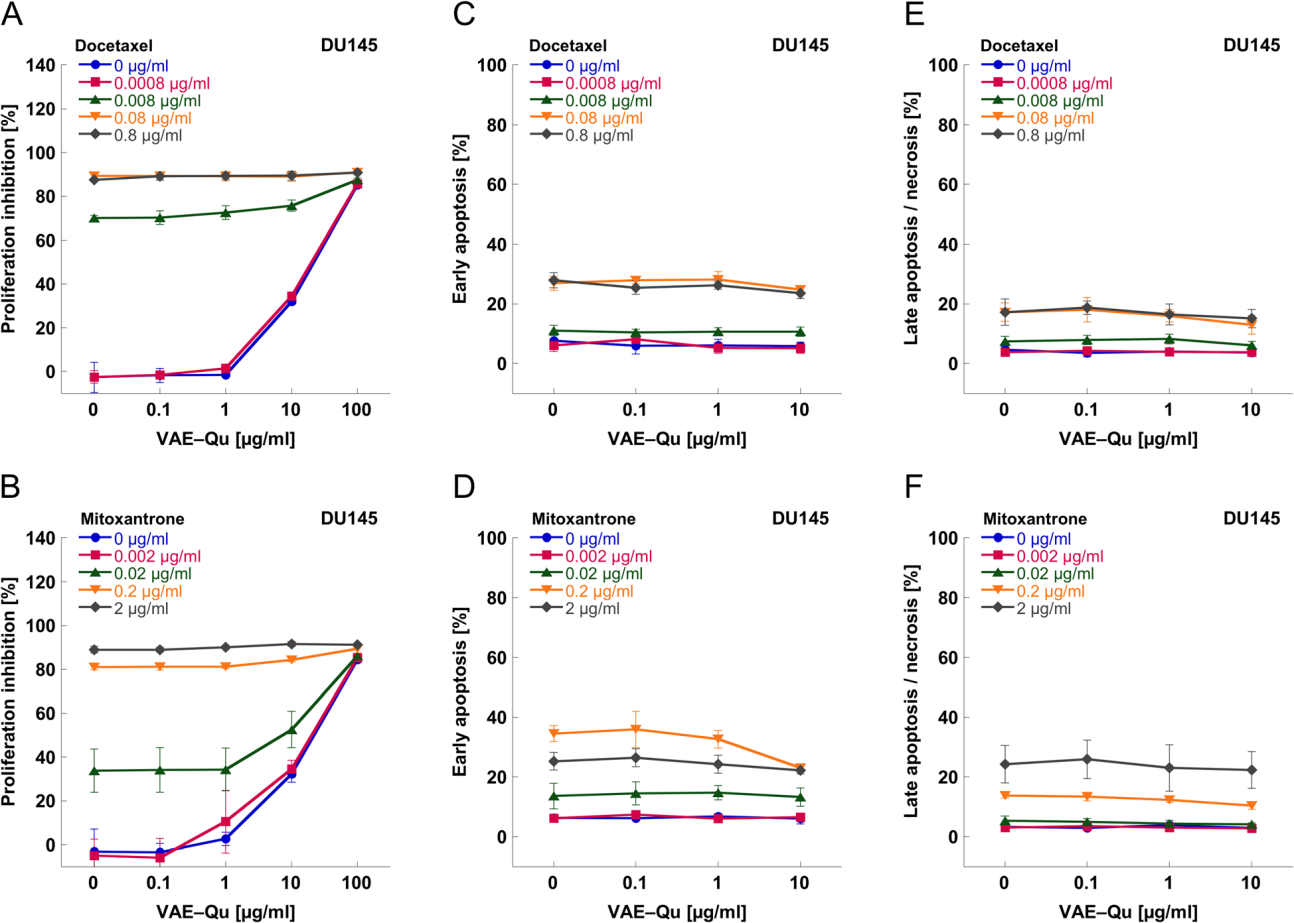
Mean values (±SD) of (A,B) proliferation, (C,D) early apoptosis and (E,F) late apoptosis/necrosis of prostate carcinoma cell line DU145 treated with different concentrations of docetaxel (A,C,E) or mitoxantrone (B,D,F), respectively combined with different concentrations of VAE-Qu.

The prostate carcinoma cell line DU145 was treated with the chemotherapeutic agents docetaxel or mitoxantrone, respectively, as well as VAE-Qu in various concentrations. The maximal cytostatic effect of all drugs applied alone was about 90%. An enforcement of chemotherapy induced cytostasis was detected at VAE-Qu concentrations of ≥10 μg/ml (p < 0.01) for medium concentrations of docetaxel (0.0008, 0.008 μg/ml) or mitoxantrone (0.002, 0.02 μg/ml) (Figure 4A,B).

Docetaxel and mitoxantrone exerted a dose dependent cytotoxic effect on DU145 cells with a maximum of about 50% cytotoxicity each (p < 0.001; Figure 4C–F). Doses between 0.1 and 10 μg/ml of VAE-Qu did not influence the cytotoxic effect of both chemotherapeutic agents (p > 0.05), with the exception of 10 μg/ml VAE-Qu at 0.2 μg/ml mitoxantrone (p < 0.05).

**Figure 5:**
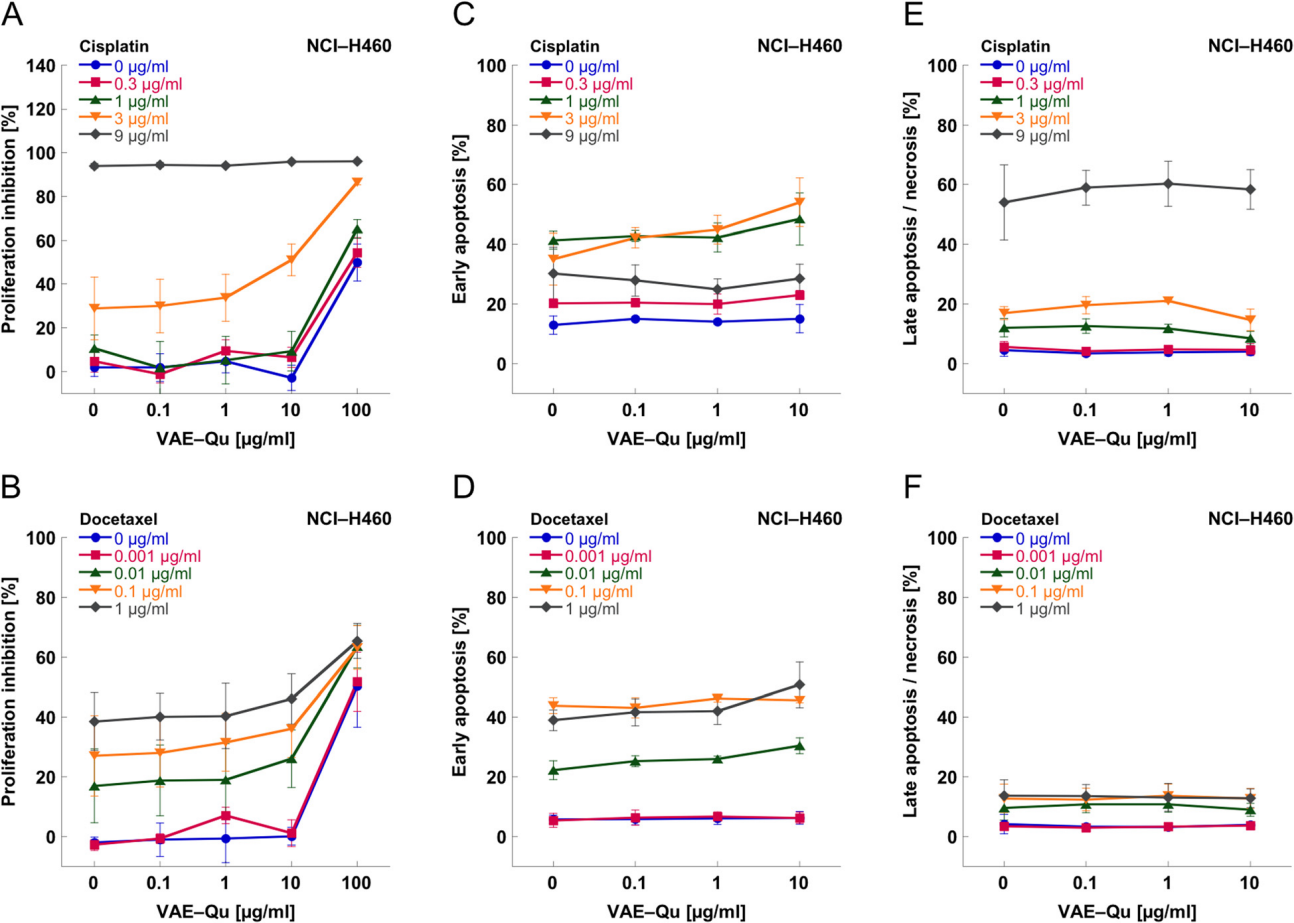
Mean values (±SD) of (A,B) proliferation, (C,D) early apoptosis and (E,F) late apoptosis/necrosis of lung carcinoma cell line NCI-H460 treated with different concentrations of cisplatin (A,C,E) or docetaxel (B,D,F), respectively, combined with different concentrations of VAE-Qu.

The treatment of the lung carcinoma cell line NCI-H460 with cisplatin at a concentration of 9 μg/ml resulted in a proliferation inhibition of 95% (Figure 5A), whilst VAE-Qu (100 μg/ml) inhibited proliferation by 50% (Figure 5A,B). The maximal cytostatic effect attained by the treatment with docetaxel (1 μg/ml) was about 40% and – as in PA-TU-8902 cells – could not further be augmented by dose enhancement (Figure 5B). Only VAE-Qu at a concentration of 100 μg/ml could additionally enhance the antiproliferative effect of docetaxel (Figure 5B), as it did for 0.3–3 μg/ml cisplatin (Figure 5A).

The dose dependent cytotoxic effect of cisplatin and docetaxel on NCI-H460 revealed a maximal cytotoxicity for cisplatin of 85% and for docetaxel of 55%. In general, no significant influence of VAE-Qu at concentrations between 0.1 and 10 μg/ml was observed; only at 3 μg/ml cisplatin, VAE-Qu 1 and 10 μg/ml additionally enhanced early apoptosis (p < 0.05, Figure 5C), as did 10 μg/ml VAE-Qu at 0.01 and 0.1 μg/ml docetaxel (p < 0.05, Figure 5D).

## Discussion

No inhibition of chemotherapy-induced cytostasis by VAE was observed in any of our experimental settings. In general, VAE at concentrations between 0.1 and 10 μg/ml neither enhanced nor decreased the amount of chemotherapy induced early and late apoptosis and necrosis. At concentrations ≥10 μg/ml, VAE led to an additive augmentation of chemotherapy induced cytostasis.

Since cancer patients receive besides anticancer agents numerous medications for supportive care and treatment of comorbid illnesses, consideration of metabolic interactions is important. Drug interactions could influence efficacy and toxicity of cytostatic drugs. For example cytotoxicity of taxanes which stabilize microtubule structures and thereby block the mitotic spindle apparatus is very susceptible to drugs that induce cell cycle arrest. Their effect can be potentiated or antagonized depending on the sequence of applied drugs [21].

Although mistletoe is frequently used in addition to conventional cancer therapeutics, there is only little information about possible interactions with chemotherapeutic drugs. Many anticancer drugs are metabolized by cytochrome P isoenzymes (CYPs) and the metabolism and pharmacokinetics of anticancer agents may be altered by herbal medicines. Thus, inhibition of CYPs could affect the intracellular concentration of drugs. Mistletoe was reported to be an inhibitor of CYP3A4 in vitro [22], however, the corresponding IC_50_ values are physiologically irrelevant. The investigation of interferences of mistletoe with cytochrome P450 isoforms in human hepatocytes indicated no or only minor potential for herb-drug interactions [23], suggesting that clinically significant systemic interaction is unlikely.

The aim of our study was to investigate if clinically relevant doses of VAE interfere with standard chemotherapeutic agents in vitro by influencing their cytostatic and cytotoxic efficacy. We used the standard chemotherapeutic drugs doxorubicin for the treatment of breast cancer cell lines HCC1141 and HCC1937 [24], gemcitabine for the treatment of pancreatic carcinoma cell line PA-TU-8902 [25], mitoxantrone and docetaxel for the treatment of prostate cancer cell line DU145 [26] and cisplatin and docetaxel for the treatment of lung carcinoma cell line NCI-H460 [27]. According to typical usage in integrative oncological settings, Iscador M spec. (VAE-M) was used for the treatment of breast and Iscador Qu spec. (VAE-Qu) for the treatment of pancreatic, prostate and lung cancer cell lines [19,28,29].

Initially analyzing a sole VAE application we could demonstrate the well known anti-proliferative effects of higher doses of mistletoe extracts on cancer cell lines. The direct anti-proliferative and cytotoxic activity of mistletoe is based mainly on a dose dependent apoptotic effect of mistletoe lectins (ML) [30] which in case of ML I requires the internalization of its A chain that inactivates the 28 S ribosomal subunit leading to inhibition of protein synthesis and to induction of apoptosis via the intrinsic pathway [31-33]. Growth inhibition by mistletoe may also be the result of a cell cycle blockade in G0/G1 phase [34]. High concentrations of ML and viscotoxins cause cell lysis mainly through necrosis [35,36].

In the context of supportive therapy with chemotherapy protocols, where no direct induction of tumor-cell specific apoptosis by mistletoe is intended, patients usually are treated with VAE doses between 0.01 and 20 mg by 2 to 3 weekly subcutaneous injections. The concentrations of 0.1 and 1 μg/ml VAE are roughly corresponding to an injection of 5 mg Iscador when referring to the amount of circulating blood or body weight, respectively. Our results show that these lower, clinically typical VAE doses influenced neither proliferation nor apoptosis of the investigated cell lines.

VAE concentrations ≥10 μg/ml partially had an additive effect on chemotherapy induced cytostasis. Additive effects were previously shown in highly ML-sensitive Jurkat cells, where very low nontoxic concentrations of purified ML-I markedly enhanced etopside-induced apoptosis [33]. Siegle et al. demonstrated additive cytotoxic activity of Viscum album agglutinin-I (VAA-I) in combination with doxorubicin, cisplatin and taxol in the human lung carcinoma cell line A549 [37].

*In vitro* determination of cytostasis or cytotoxicity depends on assay conditions like doses used, incubation time and the cellular context. In our experiments, the cytostatic effects distinctly exceeded the cytotoxic effects for the chemotherapeutic agents and VAE alone or in combination. Most of the conventional anticancer agents are both cytostatic and cytotoxic [1]. Cytostasis can be the initial step for different mechanisms of cell death whereby the duration of mitotic arrest does not necessarily correlate with the probability of death [38]. In apoptosis-sensitive cell lines, prolonged mitotic arrest induced by antimitotic drugs causes apoptosis. In less sensitive cell lines, cells undergo slippage without division into tetraploid G1, which may be followed by p53-dependent arrest, apoptosis, or another round of mitosis [38-41]. On the other hand it is well known that mutations in the apoptotic program (i.e. p53 mutations) and up-regulated pro-survival signals (anti-apoptotic Bcl2 family members) in established cancers contribute to resistance to apoptotic cell death and are important aspects of resistance to anticancer therapies [42,43].

Iscador adjuvant to chemotherapy was reported to decrease therapy-related adverse drug reactions, to increase response rates and to improve disease symptom control, quality of life and overall survival [15,18,44,45]. *In vitro* and *in vivo* studies revealed several effects that may contribute to explain the mistletoe related clinical benefits. In cyclophosphamide exposed cells *in vitro*, mistletoe extracts exerted a protective effect on peripheral mononuclear cells (PBMC) from healthy donors but not on malignant Jurkat leukemia cells by the enhancement of mitochondrial activity and replication [46]. In PBMC, mistletoe extracts improved DNA repair of damaged cells [47] and reduced sister chromatide exchange [48]. Numerous effects of mistletoe extracts on the immune system are known [11]. It is hypothesized that these immunomodulating properties augment systemic antitumor effects and contribute to a reduction of chemotherapy-associated immune suppression.

Cancer cell lines have been widely used to study the biological mechanisms involved in cancer and to examine the factors influencing the response of tumors to therapeutic agents and regimens. In general, cancer cell lines show similar morphologic and molecular characteristics of the primary tumor and maintain the expression of most cancer characteristics. However, they also have a major disadvantage. Cells are removed from their natural environment and interaction and protection mechanisms otherwise available from the donor organism are eliminated. Cancer cell lines often originate from aggressive and metastatic tumors and may not properly reflect the situation in earlier stage and lower grade disease. These factors must be considered when interpreting the results of our study.

Testing the effect of mistletoe extracts on chemotherapeutics *in vitro* with a limited number of cell lines and test substances is a basic step in completing the knowledge about possible herb-drug interactions and cannot replace clinical investigations.

## Conclusions

Aqueous, fermented mistletoe extracts did not influence the cytostatic and cytotoxic activity of several common conventional chemotherapeutic drugs when applied in concentrations typical for clinical use. We could show this in breast, prostate, pancreatic and lung carcinoma cell lines. Although these *in vitro* data cannot directly be extrapolated to the complex *in vivo* conditions, they contribute to the knowledge regarding safety of cancer patients receiving mistletoe supported chemotherapy. Our *in vitro* results are in line with clinical experiences and trials that Iscador can be used concomitant with conventional oncological drugs without safety hazard by herb drug interactions.

## Competing interests

We received limited funding for scientific projects by the manufacturer, who, however, had no influence on the analysis, or interpretation of data, on the writing of manuscripts, or on the decision to submit the manuscripts for publication.

## Authors’ contributions

UW and MK made substantial contributions to the conception and design of the study and performed analyses, SB performed statistical analysis, UW drafted, and KU and SB critically revised the manuscript. All authors read and approved the final manuscript.

## Pre-publication history

The pre-publication history for this paper can be accessed here:

http://www.biomedcentral.com/1472-6882/14/6/prepub
